# Guillain-Barre syndrome: a typical paraneoplastic syndrome in a kidney transplant recipient with allograft renal cell carcinoma: a case report and review of the literature

**DOI:** 10.1186/s12882-020-02095-y

**Published:** 2020-10-14

**Authors:** Izabela Zakrocka, Iwona Baranowicz-Gąszczyk, Agnieszka Korolczuk, Wojciech Załuska

**Affiliations:** 1 Department of Nephrology, Medical University, Jaczewskiego street 8, 20-090 Lublin, Poland; 2 Department of Clinical Patomorphology, Medical University, Lublin, Poland

**Keywords:** Guillain-Barré syndrome, Kidney, Kidney transplantation, Papillary renal cell carcinoma

## Abstract

**Background:**

Guillain-Barré syndrome (GBS) is an autoimmune polyneuropathy affecting the peripheral nervous system. This neurological disorder has been previously reported in bone marrow transplant recipients but is uncommon after kidney transplantation. Viral infections and calcineurin inhibitors are the main triggers of GBS in renal transplant recipients.

**Case presentation:**

In this report, we present a case of a 47-year-old male patient 12 years after his second kidney transplantation who developed GBS due to papillary renal cell carcinoma. Infectious and drug-related origins of GBS were excluded. Despite intensive treatment, graftectomy was performed, after which neurological symptoms resolved.

**Conclusions:**

In kidney transplant recipients, paraneoplastic aetiology should be considered in the differential diagnosis of GBS.

## Background

Neurological complications remain one of the biggest challenges among patients after solid organ transplantation. It is estimated that nervous system dysfunction occurs in 9 out of 10 transplanted patients but is often not reported [1]. Among symptoms experienced by renal transplant recipients, those connected with peripheral nerve damage are scarce. Guillain-Barré syndrome (GBS) is the most common acute ascending polyneuropathy in adults, affecting 0.5–2 per 100,000 people per year [2]. The majority of reported cases after transplantation are composed of men with symptoms occurring from 2 days to 10 years after graft implantation. Unlike bone marrow transplant recipients, GBS in solid organ transplant patients is less often diagnosed. GBS after transplantation is predominantly linked with infections caused by cytomegalovirus (CMV) [3], Epstein-Barr virus (EBV) [4], *Campylobacter jejuni* and calcineurin inhibitors [2]. The paraneoplastic aetiology of peripheral polyneuropathy due to renal cell carcinoma [5, 6] or its treatment [7] was previously reported but not in renal transplant recipients.

## Case presentation

**Fig. 1:**
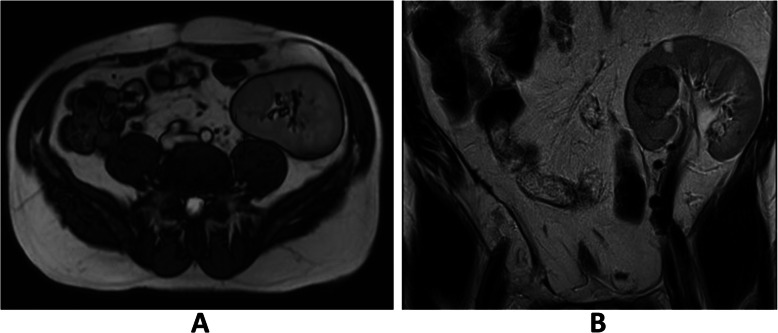
**a**. Magnetic resonance imaging of the abdomen: tumour in the cortex of the transplanted kidney. Turbo spin echo (TSE), fat suppression sequence, transverse plane. **b**. Magnetic resonance imaging of the abdomen: tumour in the cortex of transplanted kidney. Cortical cyst in the upper pole of the transplanted kidney. Turbo spin echo (TSE), fat suppression sequence, frontal plane

**Fig. 2:**
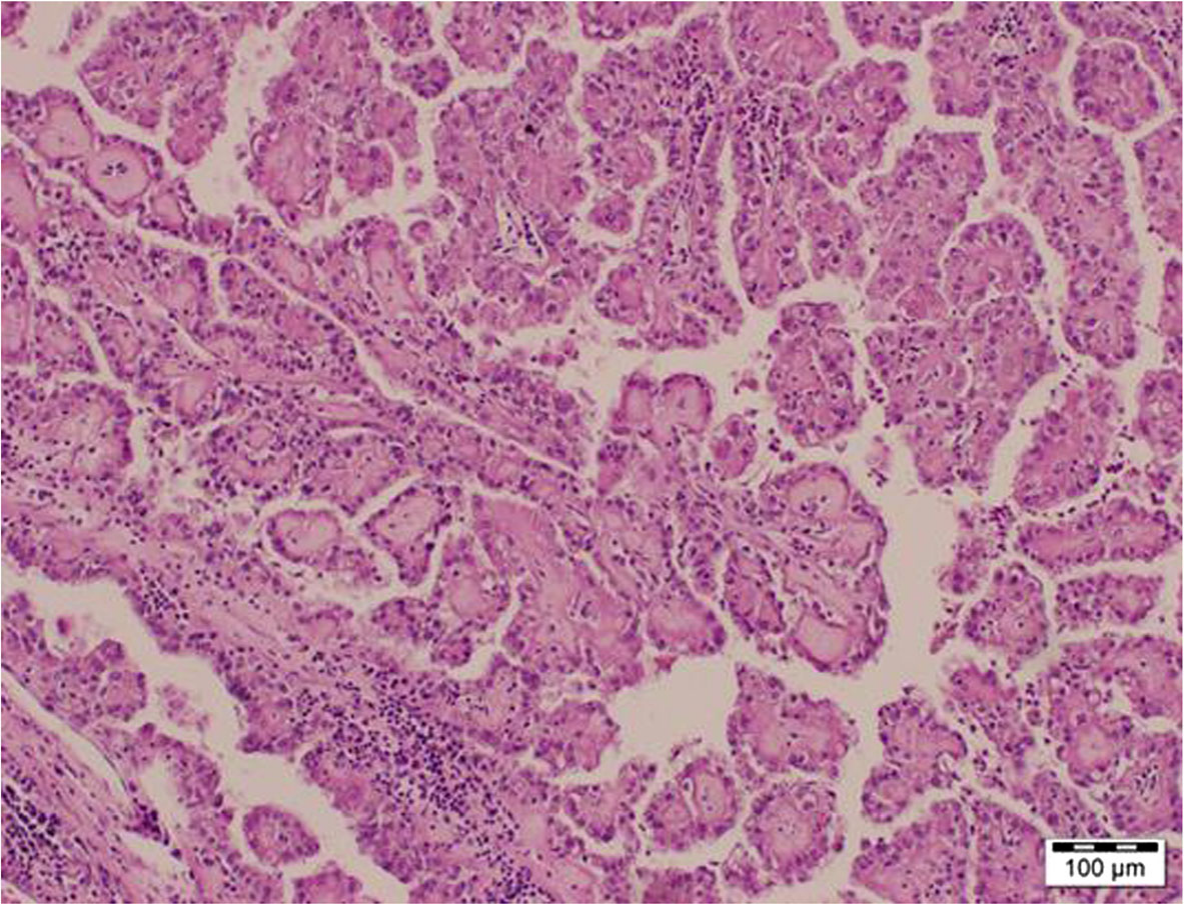
Papillary renal cell carcinoma (type 2). Fibrovascular cores covered by pseudostratified eosinophilic neoplastic epithelium. Haematoxylin and eosin staining

**Fig. 3:**
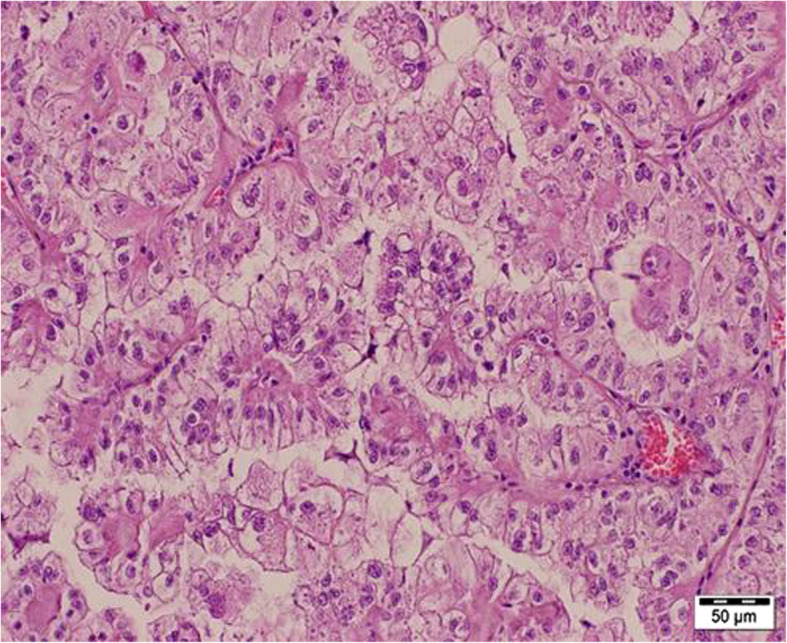
Papillary renal cell carcinoma (type 2). Papillary structures covered by eosinophilic neoplastic cells with mild nuclear pleomorphism and visible nucleoli (International Society of Urologic Pathologists/World Health Organization grading system Grade 3). Haematoxylin and eosin staining

**Fig. 4:**
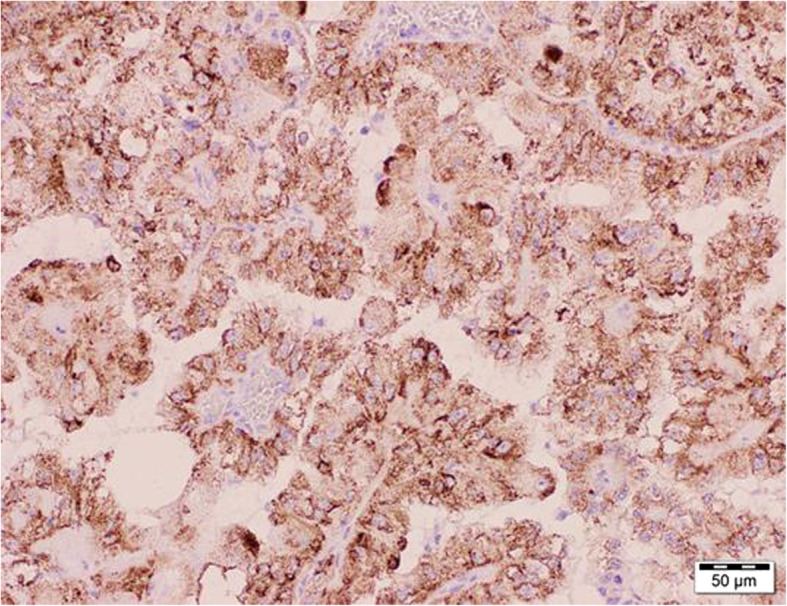
Papillary renal cell carcinoma (type 2). Strongly positive immunohistochemical staining for α-methylacyl CoA racemase (AMACR)

**Fig. 5:**
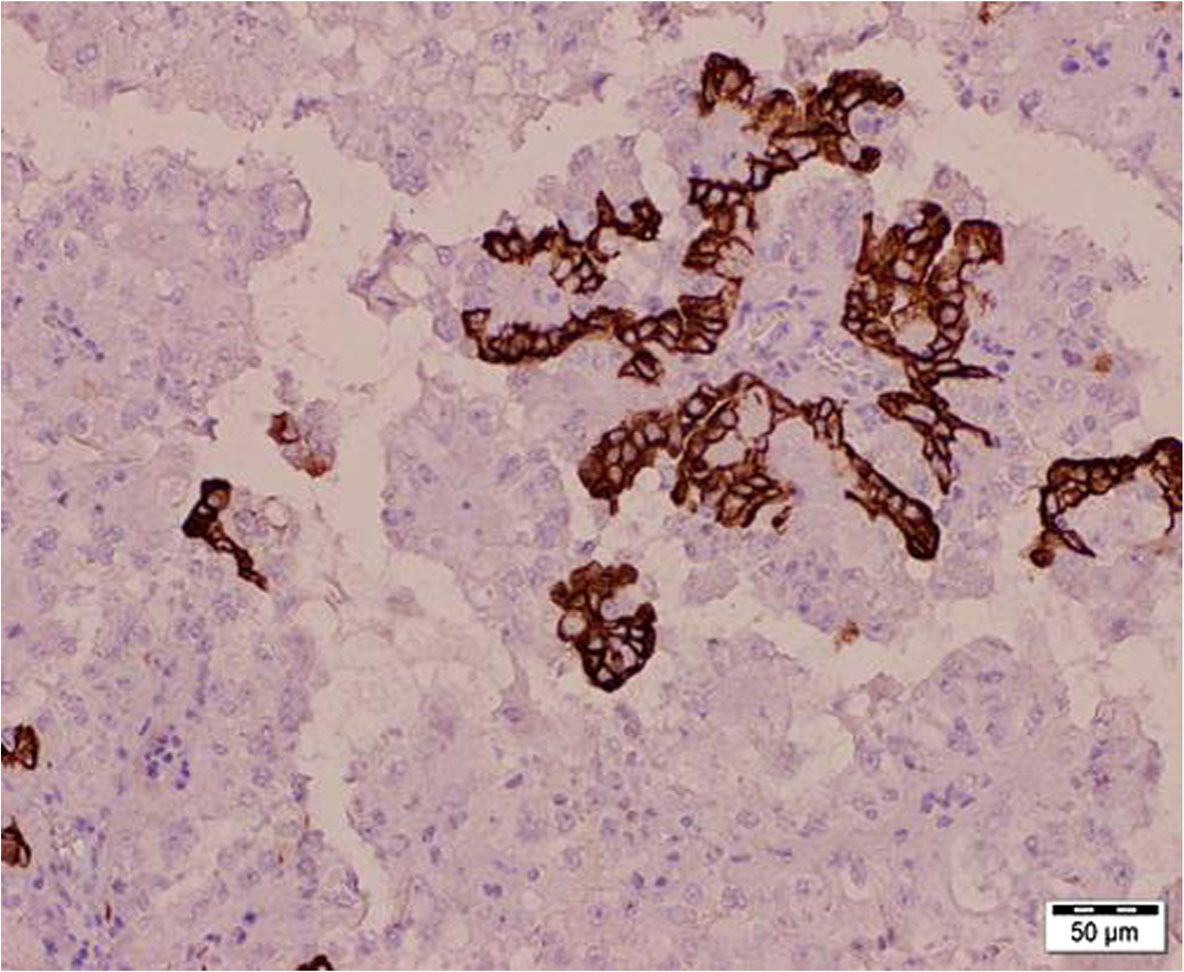
Papillary renal cell carcinoma (type 2). Focal positive immunohistochemical staining for cytokeratin 7 (CK 7)

A 47-year-old man with a history of chronic kidney disease since 1986, probably due to glomerulonephritis (kidney biopsy was not performed), was admitted to the Neurology Department in February 2014, 12 years after a second kidney transplantation from a deceased donor, because of paresthesia, numbness and symmetric progressive weakness of the lower limbs lasting for 3 weeks. First, kidney transplantation was performed in 1987 and was complicated with urinary fistula. The patient did not receive induction immunosuppression and received prednisone with azathioprine for maintenance treatment. Renal transplant was lost in the mechanism of antibody-mediated rejection, and graftectomy was performed in 1992. The right native kidney was removed in 1995 because of hydronephrosis. After the second kidney transplantation (2002), graft function was stable (serum creatinine 1.1 mg/dl, estimated glomerular filtration rate [eGFR] 74.3 ml/min/1.73 m^2^), and the haemoglobin concentration was within the normal range (17.1 g/dl – 17.4 g/dl; laboratory limit 18.0 g/dl). For immunosuppression after the second transplantation, the patient received mycophenolate mofetil with cyclosporine and prednisone, and no antibody induction was used. A year before neurological symptoms started to occur, abdominal ultrasound revealed a single cortical cyst 9 mm in diameter in the graft, whereas the left native kidney was not visualized. During hospitalization in the Neurology Department, symmetric paralysis of the lower limbs and areflexia were confirmed together with distal paralysis of the upper limbs. Abnormalities in superficial and deep sensation as well as pyramidal signs were not observed. The nerve conduction study revealed peripheral nerve damage, with local motor conduction blockade, slower nerve conduction in the popliteal area to 20–16.6 m/s and prolonged latency to 250% of the normal value. Lumbar puncture was performed, and in the cerebrospinal fluid, protein-cytological dissociation was confirmed (protein concentration 127 mg/dl, cytosis 3 cells/μl). During the observation period, the patient’s body temperature was normal, and gastrointestinal, urinary and respiratory symptoms were not observed. In the laboratory tests, the white blood cell (WBC) count was 9.41 × 10^3^/μl with 83.0% neutrophils and 11.1% lymphocytes, the haemoglobin level was 17.3 g/dl, C-reactive protein (CRP) was 0.737 mg/l, erythrocyte sedimentation rate (ESR) was 4 mm/h, procalcitonin (PCT) was 0.04 ng/ml, serum urea was 49.3 mg/dl, and serum creatinine was 1.1 mg/dl (eGFR 75.0 ml/min/1.73 m^2^). Cyclosporine levels were in the range of 107–125 ng/ml. The patient was EBV IgG (immunoglobulin G)-positive and EBV IgM (immunoglobulin M)-negative. Similarly, CMV IgG was present, whereas CMV IgM was not found in the patient’s serum. CMV, EBV, *Borrelia burgdorferi*, hepatitis B virus (HBV) and hepatitis C virus (HCV) active infections were excluded by polymerase chain reaction (PCR) tests. Urine analysis revealed no abnormalities. In the ultrasound of the abdomen, a round-shaped normoechogenic mass 34x34x28 mm in size in the cortex of transplanted kidney was found. Doppler ultrasound of the transplanted kidney revealed a lack of signal in this area, which led to a suspicion of malignancy. In abdominal magnetic resonance imaging (MRI), except for two cortical cysts (10 mm and 3 mm in diameter), a well-organized tissue area in the transplanted kidney cortex was shown, providing more evidence of a neoplastic process (Fig. 1a, b). Due to severe neurological symptoms, the patient presented three procedures of therapeutic plasma exchange (TPE). The patient’s neurological condition after TPEs slightly improved. However, due to transplanted kidney malignancy suspicion, partial graftectomy was planned. Unfortunately, during surgery, a kidney tumour with multifocal infiltration of the renal medulla was found, so a decision was made to perform total graftectomy. Histopathological examination of the kidney revealed an encapsulated, solid tumour measuring 3 cm in diameter, located within the upper pole of the kidney, infiltrating the renal capsule and medulla. Microscopic examination showed papillary renal cell carcinoma (type 2) with papillary folds covered with eosinophilic mildly pleomorphic cells (Fig. 2). Nucleoli were visible (Fig. 3). Immunohistochemistry showed tumour cells positive for cluster of differentiation 10 (CD 10) and α-methylacyl CoA racemase (AMACR) (Fig. 4) and only focally positive for cytokeratin 7 (CK7) (Fig. 5), which excluded clear cell papillary renal cell carcinoma (World Health Organization grading system 2017) in the differential diagnosis. After graftectomy, no tumour reoccurrence or metastases were observed, and the patient’s neurological symptoms resolved. Intermittent haemodialysis was started. Six years later, the patient’s neurological status was normal. Since no abnormalities in laboratory or radiological tests were observed and the oncological grace period expired, the patient qualified for third kidney transplantation.

## Discussion and conclusions

GBS is a demyelinating polyneuropathy with a mortality rate estimated from 3 to 8%, rising to 15–30% in patients requiring mechanical ventilation [8], while approximately 20% of patients can be left with severe disability [9]. Due to different clinical courses, GBS is divided into two categories: acute inflammatory demyelinating polyneuropathy (AIDP) and acute motor axonal neuropathy (AMAN). In Western countries, AIDP is the most common manifestation of GBS [10]. Molecular mimicry of bacterial or viral molecules by the human gangliosides on the motor axolemma is claimed to be responsible for AMAN [11]. In contrast, the pathogenesis of AIDP is less understood, with possible complement activation leading to Schwann cell damage and demyelination [12]. In our case, the patient presented mixed sensory and motor dysfunction and was considered to have AIDP.

Given that GBS is a neurological manifestation of autoimmune processes, patients often do not develop GBS after bone marrow or solid organ transplantation. Broad spectrum immunosuppressive regimens, especially those containing mycophenolate mofetil (MMF), seem to be protective against GBS [9]. Inhibition of B cell proliferation and differentiation resulting in lower antibody production are the main beneficial effects of MMF [11]. Moreover, the reduction of mononuclear cell recruitment to inflammatory sites and nitric oxide production are other MMF mechanisms protecting against GBS [11]. However, GBS was also reported in patients with acquired immunodeficiency syndrome and severe T-cell suppression [13]. Additionally, GBS can be evoked by administration of antilymphocyte globulin [14] or calcineurin inhibitors, cyclosporine [15] and tacrolimus [16], due to inappropriate T cell inhibition [8]. Despite many reports, the role of calcineurin inhibitors in GBS pathogenesis seems intriguing. There are some observations suggesting a therapeutic effect of cyclosporine in patients with GBS [17, 18]. Our patient received a similar immunosuppressive regimen for 12 years: mycophenolate mofetil, cyclosporine and prednisone, and the patient had stable serum drug concentrations without the need for immunosuppressive therapy modification.

Most cases of GBS have been reported in bone marrow transplant recipients and are associated with graft versus host disease (GvHD) [19]. In recipients of solid organ transplants, mainly the heart, liver and kidney, GBS usually appears within 1 year after transplantation and is mainly related to CMV infection [2]. According to the patient’s CMV and EBV serostatus in the present study, tissue invasion cannot be fully excluded; however, no obvious infectious cause of GBS was found in the performed laboratory tests. Late onset of symptoms after kidney transplantation related to the occurrence of transplanted kidney tumour and neurological improvement after graftectomy indicated a paraneoplastic aetiology of GBS.

In publicly available data, clear cell renal carcinoma, as the most common histological type of renal carcinoma, was related to GBS in nontransplant patients (Table 1).

**Table 1:** Characteristics of GBS cases associated with renal cell carcinoma

Type of renal cell carcinoma	Clinical manifestation	Time of GBS onset before diagnosis	Reference
Papillary	paresthesia, numbness and symmetric progressive weakness of lower limbs	3 weeks	Presented manuscript
Clear cell	progressive gait disturbance and muscle weakness	one month	Nishioka K et al. [5]
Clear cell	progressive weakness, sensory changes, and urinary retention	one year	Yang I et al. [6]
Papillary	numbness bilaterally in her feet, hands, and lips; difficulties with balance and manipulating objects with her hands	three months	Allen JA et al. [20]
Clear cell	muscle weakness and fasciculations in the upper extremities	five months	Turk HM et al. [21]
Clear cell	diplopia, dysarthria, dysphagia, and bilateral lower extremity weakness	“fulminant”, not specified	Roy MJ et al. [22]
Clear cell	facial palsy and progressive weakness of both arms and legs	not specified, month since symptoms aggravated	Alimonti A et al. [23]
Not specified	progressive respiratory and limb muscle weakness	three weeks	Forman D et al. [24]
Not specified	atypical, progressive neuropathy after nephrectomy	two months	Kim et al. [25]
Not specified	motor neuron disease	not specified	Evans et al. [26]

Among reported GBS in only one case, a relationship between papillary renal carcinoma and GBS was presented [20]. Interestingly, in a study presented by Kim et al., nephrectomy due to renal carcinoma was claimed to be a trigger of GBS [25]. Patients with cancer-related GBS showed various symptoms. Progressive weakness of limbs was accompanied by gait disturbance, urinary retention, facial palsy, diplopia, dysarthria, and dysphagia. The time from symptom onset until GBS diagnosis was also different, varying from weeks to months (Table 1). Nonspecific and long-lasting symptoms in most cases delayed appropriate diagnosis. Moreover, most neurological complications in transplant patients are thought to be related to immunosuppressive agents or opportunistic infections, leading to the underreporting of cancer-related GBS. Gentile et al. presented a case of a patient 7 years after kidney transplantation who developed acute complete bilateral ophthalmoplegia, areflexia of all four limbs and gait ataxia due to Burkitt’s lymphoma of the graft [27]. In our study and that by Gentile et al. [27], the occurrence of GBS was related to late kidney neoplasm occurrence since cancer risk is known to increase with the time after transplantation [28]. Despite low 20-year incidence rates, genitourinary cancers remain one of the most common cancers diagnosed after renal transplantation [28].

Moreover, a higher haemoglobin concentration did not exceed the upper limit in our laboratory of 18.0 g/dl, which could be another marker of paraneoplastic activity. Posttransplant erythrocytosis (PTE) occurs mainly in men with their native kidneys and without a history of graft rejection [29]. Among other risk factors for PTE are smoking, adequate erythropoiesis prior to transplantation, renal artery stenosis, polycystic kidney disease and renal cell carcinoma [30]. PTE usually occurs up to 24 months after transplantation in 19–22.2% of patients [29, 30]. Within the years following transplantation, PTE subsides due to worsening of graft function [29]. If PTE persists, the goal of the treatment is to lower the haemoglobin level below 17.5 g/dl [30] with the use of angiotensin-converting enzyme inhibitors (ACE-I), angiotensin II type 1 receptor blockers (ARB), theophylline or phlebotomy [31]. In the present case, the patient’s haemoglobin level remained high beginning 1 year after transplantation and did not exceed 17.5 g/dl due to telmisartan administration; however, it was given in small doses because of low blood pressure values.

Notably, malignancies after kidney transplantation are predominantly reported in native kidneys [32]. Papillary renal cell carcinoma remains the most common type of cancer in patients with advanced kidney disease receiving haemodialysis [33]. Atypical localization and histological type of renal carcinoma found in our patient after kidney transplantation highlight the need for diagnostic awareness in patients presenting GBS symptoms.

In the present study, an extremely rare form of GBS caused by renal transplant carcinoma is reported. Potentially paraneoplastic aetiology of GBS should be excluded in every patient after transplantation.

## Data Availability

The datasets used and analysed during the current study are available from the corresponding author on reasonable request.

## Abbreviations

ACE-I: Angiotensin-converting enzyme inhibitors
AIDP: Acute inflammatory demyelinating polyneuropathy
AMACR: Α-methylacyl CoA racemase
AMAN: Acute motor axonal neuropathy
ARB: Angiotensin II type 1 receptor blockers
CD 10: Cluster of differentiation 10
CK 7: Cytokeratin 7
CMV: Cytomegalovirus
CRP: C-reactive protein
EBV: Epstein – Barr virus
eGFR: Estimated glomerular filtration rate
ESR: Erythrocyte sedimentation rate (ESR)
GBS: Guillain-Barré syndrome
GvHD: Graft versus host disease
HBV: Hepatitis B virus
HCV: Hepatitis C virus
IgG: Immunoglobulin G
IgM: Immunoglobulin M
MMF: Mycophenolate mofetil
MRI: Magnetic resonance imaging
PCR: Polymerase chain reaction
PCT: Procalcitonin
TPE: Therapeutic plasma exchange
TSE: Turbo spin echo
WBC: White blood cells

## References

[1] Piotrowski PC, Lutkowska A, Tsibulski A, Karczewski M, Jagodziński PP (2017). Neurologic complications in kidney transplant recipients. Folia Neuropathol.

[2] Ostman C, Chacko B (2019). Guillain-Barré syndrome post renal transplant: A systematic review. Transpl Infect Dis.

[3] Keithi-Reddy SR, Chakravarthi RM, Hussaini SM, Venkatapuram RR, Murthy JM (2007). Cytomegalovirus disease with Guillain-Barré syndrome in a cadaver renal allograft recipient: cause or coincidence. Int Urol Nephrol.

[4] Masajtis-Zagajewska A, Muras K, Mochecka-Thoelke A, Kurnatowska I, Nowicki M (2012). Guillain-Barré syndrome in the course of EBV infection after kidney transplantation-a case report. Ann Transplant.

[5] Nishioka K, Fujimaki M, Kanai K, Ishiguro Y, Nakazato T, Tanaka R, Yokoyama K, Hattori N (2017). Demyelinating peripheral neuropathy due to renal cell carcinoma. Intern Med.

[6] Yang I, Jaros J, Bega D (2017). Paraneoplastic Peripheral Nervous System Manifestations of Renal Cell Carcinoma: A Case Report and Review of the Literature. Case Rep Neurol.

[7] Koshikawa H, Tsukie T, Kurita A, Fujikura M, Suzuki M, Araki K (2017). Guillain-Barré syndrome in a patient with renal cell carcinoma following the first course of pazopanib therapy. J Infect Chemother.

[8] El-Sabrout RA, Radovancevic B, Ankoma-Sey V, Van Buren CT (2001). Guillain-Barré syndrome after solid organ transplantation. Transplantation..

[9] Shaban E, Gohh R, Knoll BM (2016). Late-onset Cytomegalovirus infection complicated by Guillain-Barre syndrome in a kidney transplant recipient: case report and review of the literature. Infection..

[10] Kuwabara S (2007). Guillain-barré syndrome. Curr Neurol Neurosci Rep.

[11] Suthanthiran M, Morris RE, Strom TB (1996). Immunosuppressants: cellular and molecular mechanisms of action. Am J Kidney Dis.

[12] Hafer-Macko CE, Sheikh KA, Li CY, Ho TW, Cornblath DR, McKhann GM, Asbury AK, Griffin JW (1996). Immune attack on the Schwann cell surface in acute inflammatory demyelinating polyneuropathy. Ann Neurol.

[13] Qureshi AI, Cook AA, Mishu HP, Krendel DA (1997). Guillain-Barré syndrome in Immunocompromised patients: a report of three patients and review of the literature. Muscle Nerve.

[14] Kaya B, Davies CE, Oakervee HE, Silver NC, Gawler J, Cavenagh JD (2005). Guillain Barré syndrome precipitated by the use of Antilymphocyte globulin in the treatment of severe aplastic Anaemia. J Clin Pathol.

[15] Falk JA, Cordova FC, Popescu A, Tatarian G, Criner GJ (2006). Treatment of Guillain-Barré syndrome induced by cyclosporine in a lung transplant patient. J Heart Lung Transplant.

[16] Meena P, Bhargava V, Rana DS, Bhalla AK, Gupta A, Malik M, Gupta A, Tiwari V. Tacrolimus-associated Guillain-Barre syndrome. Am J Ther. 2020. 10.1097/MJT.0000000000001096. 10.1097/MJT.0000000000001096 32091420

[17] Müllges W, Ringelstein EB, Sommer C, Biniek R, Glöckner WM (1991). Immunotherapy of chronic Guillain-Barré syndrome with high dose IgG and cyclosporin a. case report, review of the literature and perspectives. Fortschr Neurol Psychiatr.

[18] Barnett MH, Pollard JD, Davies L, McLeod JG (1998). Cyclosporin a in resistant chronic inflammatory demyelinating polyradiculoneuropathy. Muscle Nerve.

[19] Yoshida T, Ueki Y, Suzuki T, Kawagashira Y, Koike H, Kusumoto S, Ida S, Oguri T, Omura M, Sobue MN (2016). Guillain-Barré syndrome after allogeneic bone marrow transplantation: case report and literature review. eNeurologicalSci..

[20] Allen JA, Yang XJ, Sufit RL (2011). Reversible demyelinating neuropathy associated with renal cell carcinoma. Neuromuscul Disord.

[21] Turk HM, Ozet A, Kuzhan O, Komurcu F, Arpaci F, Ozturk B, Ataergin S (2009). Paraneoplastic motor neuron disease resembling amyotrophic lateral sclerosis in a patient with renal cell carcinoma. Med Princ Pract.

[22] Roy MJ, May EF, Jabbari B (2002). Life-threatening polyneuropathy heralding renal cell carcinoma. Mil Med.

[23] Alimonti A, Di Cosimo S, Di Stani F, Vecchione A, Di Palma M, Ferretti G (2003). Subacute motor weakness and left renal mass. Am J Med.

[24] Forman D, Rae-Grant AD, Matchett SC, Cowen JS (1999). A reversible cause of hypercapnic respiratory failure: lower motor neuronopathy associated with renal cell carcinoma. Chest..

[25] Kim JS, Cho JH (1992). Progressive atypical peripheral neuropathy following nephrectomy in a patient with renal cell carcinoma. J Korean Med Sci.

[26] Evans BK, Fagan C, Arnold T, Dropcho EJ, Oh SJ (1990). Paraneoplastic motor neuron disease and renal cell carcinoma: improvement after nephrectomy. Neurology..

[27] Gentile S, Messina M, Rainero I, Lo Giudice R, De Martino P, Pinessi L (2006). Miller fisher syndrome associated with Burkitt's lymphoma. Eur J Neurol.

[28] Jiang S, Regmi S, Jackson S, Calvert C, Jarosek S, Pruett T, Warlick C (2020). Risk of genitourinary malignancy in the renal transplant patient. Urology.

[29] Kolonko A, Pinocy-Mańdok J, Kocierz M, Kujawa-Szewieczek A, Chudek J, Malyszko J, Malyszko JS, Myśliwiec M, Wiecek A (2009). Anemia and erythrocytosis after kidney transplantation: a 5-year graft function and survival analysis. Transplant Proc.

[30] Malyszko J, Oberbauer R, Watschinger B (2012). Anemia and erythrocytosis in patients after kidney transplantation. Transpl Int.

[31] Vlahakos DV, Marathias KP, Agroyannis B, Madias NE (2003). Posttransplant erythrocytosis. Kidney Int.

[32] Javaid MM, Chowdhury S, Henderson A, Olsburgh J (2013). Advanced Native Kidney Renal Cell Carcinoma in Renal Transplant Recipients: Role of Sirolimus as Dual Anti-Cancer and Anti-Rejection Agent. Clin Nephrol.

[33] Billis A, Freitas LLL, Costa LBE, Barreto IS, Asato MA, Araujo KS, Losada DM, Herculiani AP, Tabosa GVBS, Zaidan BC, Oliveira GLP, Bastos LQA, Rocha RM (2017). Genitourinary malignancies in transplant or Dialysis patients: the frequency of two newly described 2016 World Health Organization Histopathologic types. Transplant Proc.

